# Cervical pre-cancerous lesion detection: development of smartphone-based VIA application using artificial intelligence

**DOI:** 10.1186/s13104-022-06250-6

**Published:** 2022-12-03

**Authors:** Ali Budi Harsono, Hadi Susiarno, Dodi Suardi, Louis Owen, Hilman Fauzi, Jessica Kireina, Rizki Amalia Wahid, Johanna Sharon Carolina, Kemala Isnainiasih Mantilidewi, Yudi Mulyana Hidayat

**Affiliations:** 1 Department of Obstetrics and Gynaecology, Faculty of Medicine, Universitas Padjajaran, Jl. Pasteur 38, Bandung, West Java 40161 Indonesia; 2 Faculty of Mathematics and Natural Sciences, Institut Teknologi Bandung, Bandung, Indonesia; 3 Biomedical Engineering, Faculty of Electrical Engineering, Telkom University, Bandung, Indonesia

**Keywords:** VIA, Cervical cancer screening, Artificial intelligence, Image processing, Low-resource settings

## Abstract

**Objective:**

Visual inspection of cervix after acetic acid application (VIA) has been considered an alternative to Pap smear in resource-limited settings, like Indonesia. However, VIA results mainly depend on examiner’s experience and with the lack of comprehensive training of healthcare workers, VIA accuracy keeps declining. We aimed to develop an artificial intelligence (AI)-based Android application that can automatically determine VIA results in real time and may be further developed as a health care support system in cervical cancer screening.

**Result:**

A total of 199 women who underwent VIA test was studied. Images of cervix before and after VIA test were taken with smartphone, then evaluated and labelled by experienced oncologist as VIA positive or negative. Our AI model training pipeline consists of 3 steps: image pre-processing, feature extraction, and classifier development. Out of the 199 data, 134 were used as train-validation data and the remaining 65 data were used as test data. The trained AI model generated a sensitivity of 80%, specificity of 96.4%, accuracy of 93.8%, precision of 80%, and ROC/AUC of 0.85 (95% CI 0.66–1.0). The developed AI-based Android application may potentially aid cervical cancer screening, especially in low resource settings.

## Introduction

Cervical cancer is the fourth most frequent cancer in women worldwide with an estimation of 604.000 new cases and 342.000 deaths in 2020. About 90% of deaths caused by cervical cancer in 2020 occurred in low- and middle-income countries [1]. The much higher incidence and mortality of cervical cancer in developing countries is mainly caused by limited access to screening programs [2, 3]. For low resource settings, WHO recommends HPV testing with treatment as screening modality. However, when HPV testing is not available, WHO recommends visual inspection with acetic acid (VIA) followed by treatment as an alternative. Apart from being cheap and easy to perform, the VIA test almost has the same sensitivity as cervical cytology (pap smear). Furthermore, VIA test allows immediate link to treatment [4–7].

Accuracy of VIA test mainly depends on the skill and proficiency of healthcare workers. Therefore, lack of comprehensive training especially in remote areas, becomes a major barrier. Digital image of cervix (cervicography) has been used to improve quality control of VIA tests. Nowadays, smartphones offer a rapid, easily accessible, cost-effective, and non-invasive way to capture these digital cervical image. With digital cervical images, VIA test result can be re-examined post-screening and can be sent to long-distance experts, thus help closing the gap in human resources. However, implementation of real-time expert consultations is still difficult due to the lack of broadband connections in remote areas [8, 9].

Recently, artificial intelligence (AI) has made its breakthrough in the world of medicine [10–12]. Artificial intelligence can automatically process images, extract features, and learn classifications through intricate algorithms [13, 14]. Automated interpretation of smartphone acquired cervical images using AI for instant VIA result prediction will help increase VIA test accuracy and enable on-site treatments to be delivered without delays.

The main objective of our study is to develop an AI-based application for determining VIA result. This development would aid health-care workers by acting as a real time decision support system, therefore extending cervical cancer screening to remote areas that lack access to experienced oncologists. To the best of our knowledge, this is the first work that develop and evaluate the performance of an AI-based VIA application used in real cervical cancer screening context in Indonesia.

## Main text

### Methods

#### Image acquisition

This study included women aged 30–50 years who were screened for cervical cancer using VIA test at Hasan Sadikin General Hospital in 2021. Informed consent was obtained from those participating in the study. Sampling was done using consecutive sampling method.

VIA test begins with the insertion of speculum and identification of squamocolumnar junction. Then, images of initial cervical conditions were taken using smartphone camera. A 3–5% acetic acid solution was then applied to the cervix. After waiting for 60 s, direct inspection of cervix was done to detect the presence of acetowhite epithelium which indicates cervical precancerous lesion. Second cervical image (after applying the acetic acid solution) was then taken. Both images (before and after acetic acid application) were sent to expert oncologist for review. Oncologist with around 20 years of professional experience, assessed these images and annotated them as positive and negative VIA results. The oncologist was kept blind to the results of the AI machine learning.

#### Artificial intelligence development

Our AI model training pipeline consists of 3 steps: image pre-processing, feature extraction, and classifier development*.*

##### Image pre-processing

Smartphone acquired VIA images contain unnecessary features, such as vaginal walls, speculum, and specular reflections. To overcome these, we first resized the images to 200 × 200 pixels size. Then, we performed specular reflection removal. We used 3 color channels from 3 color spaces, namely the saturation (S) component of HSV color space, saturation and value color space representation, green (G) component of RGB color space, and lightness (L) component of CIE-Lab color space to generate the feature image. The feature image was filtered using a standard deviation filter of size 3. Output of the filter was normalized to have values between 0 and 1. This method of specular reflections removal is inspired from [15].

**Fig. 1:**
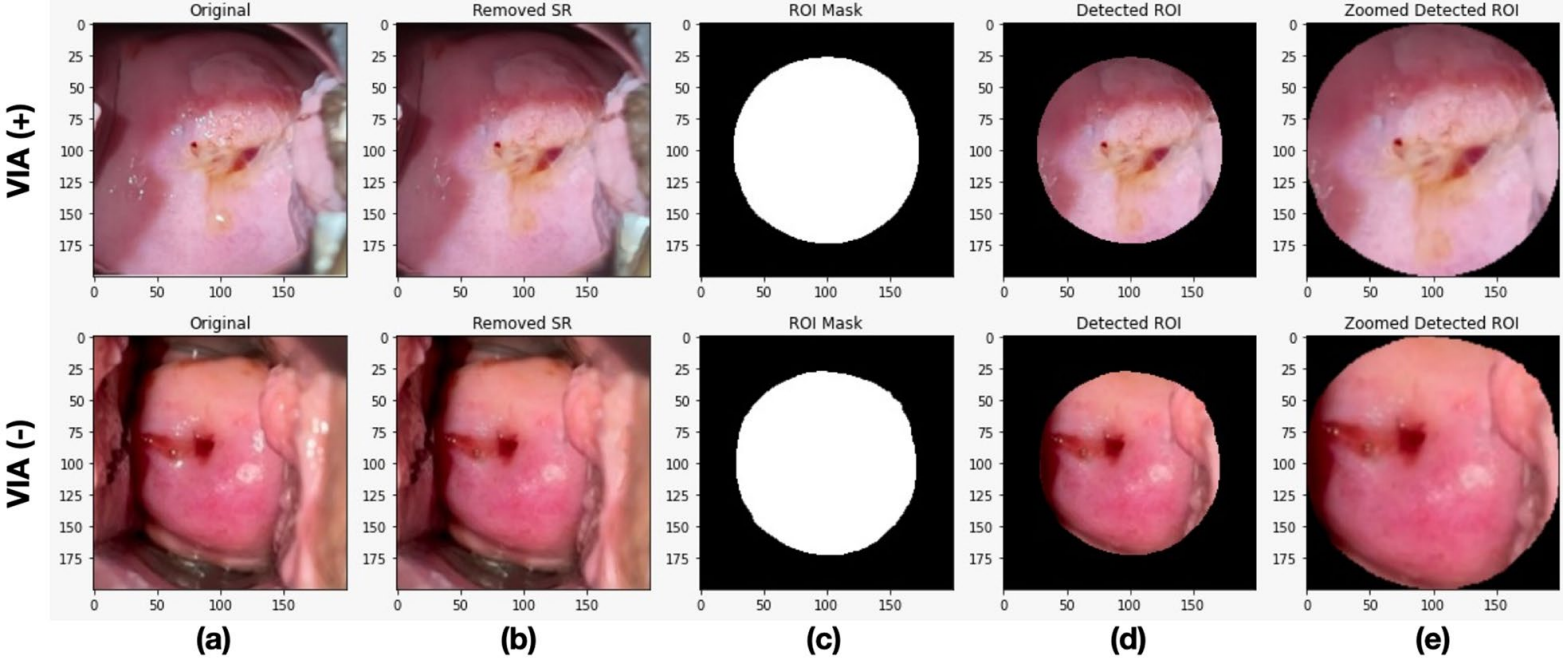
Image pre-processing steps. **a** Original image, **b** Image after specular reflections removal, **c** ROI mask generation, **d** Detected ROI, and **e** Zoomed detected ROI

Afterwards, we performed region of interest (ROI) detection using Gaussian Mixture Model (GMM) on the Ra color space where *R* is the distance of a pixel from the image center and *a* is the color channel in the CIE-Lab color space [16]. The GMM was initialized by a K-means procedure using spherical type of covariance to generate spherical-shaped ROI mask. We then zoomed in on the ROI and resized it back to 200 × 200 pixels (Fig. 1).

##### Feature extraction

Color and texture are both significant information for finding acetowhite lesions, therefore we focused on extracting these 2 group of features for classifying the images. We extracted a total of 12 color based features, namely mean and standard deviation of red (R), green (G), blue (B), green to red ratio (G/R), and blue to red ratio (B/R) of RGB color space; as well as value (V) component of HSV color space. Meanwhile texture-based features are extracted using five methods: namely Gray Level Co-occurrence Matrix (GLCM) (28 features), Neighbourhood Gray Tone Difference Matrix (NGTDM) (5 features), Gray Level Size Zone Matrix (GLSZM) (14 features), Discrete Wavelet Transform (DWT) (6 features), and Local Binary Pattern (LBP) (10 features). We then removed all the features that have high multicollinearity by using 0.9 threshold so we ended up with 31 out of 75 features in total. We also performed z-score normalization in these 31 features. It is worth to be noted that we only extracted all of these features within the detected ROI.

##### Classification

We randomly split the dataset into train-validation and test sets. We tried nine traditional machine learning algorithms as the classifier, namely Gradient Boosting Classifier, Logistic Regression, Random Forest Classifier, Extra Trees Classifier, Naive Bayes, Ada Boost Classifier, Light Gradient Boosting Machine, K Neighbors Classifier, and Decision Tree Classifier. We then performed 25-folds cross-validation on the train-validation set to choose the best algorithm using PrecisionAtRecall (0.8) as the optimization metric. PrecisionAtRecall (0.8) is a metric that computes the best precision score when recall is greater than or equal to 0.8. The chosen algorithm was Gradient Boosting Classifier (Table 1). The performance metrics used were system accuracy level, sensitivity, specificity, precision, and receiver operating characteristic (ROC) curves with area under the curve (AUC).

**Table 1:** Comparative analysis of performance of the 9 algorithms chosen

Algorithms	PrecisionAtRecall (0.8)
Gradient Boosting Classifier	0.6827
Logistic Regression	0.6613
Random Forest Classifier	0.6467
Extra Trees Classifier	0.6393
Naive Bayes	0.6160
Ada Boost Classifier	0.5880
Light Gradient Boosting Machine	0.5793
K Neighbors Classifier	0.5027
Decision Tree Classifier	0.3567

##### Tech stacks

Development of the model was done using Python programming language. We used OpenCV, PIL, Scikit-Image, Scipy, and Numpy for image processing related tasks. Pyfeats was the main package utilized for feature extraction. As for modelling, we used PyCaret, Pandas, and Scikit-learn.

#### Building the application

This Android application (named IVANET) was developed using Java programming language. Deployment of the machine learning model was done using ONNX runtime. The application enables users to input cervical image and obtain the AI model’s prediction result.

**Fig. 2:**
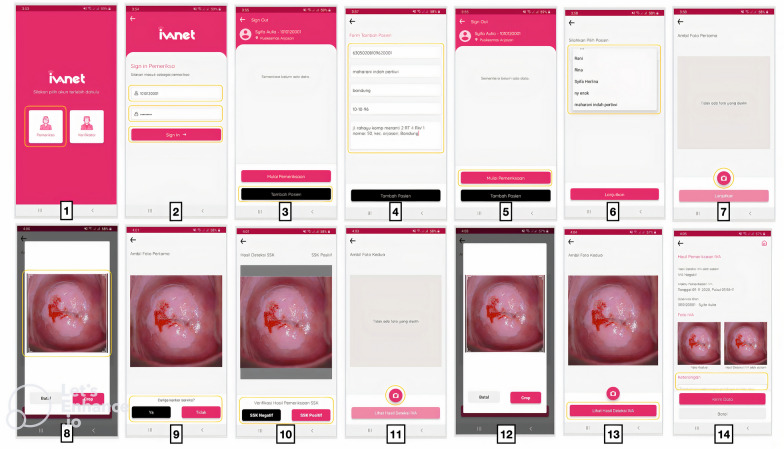
Screenshots representing IVANET Android based application input and output flow

The interface design consist of two categories: examiner and verifier. The users will first have to enter their code and password to sign in. In the examiner’s dashboard, users will be presented with all the registered patients list. The user will also be given a choice to add or register a new patient. To start the process, the user will have to select the preferred patient’s name, then click on the camera button to upload or capture the first cervical image (before VIA test). The user will then be asked to manually crop the picture around the cervical region. After that, the users have to confirm whether the picture taken is suspicious of cervical cancer or not. If there are no obvious signs of cervical cancer, the user will then be asked to manually determine the presence of SCJ by either choosing the “Positive SCJ” or “Negative SCJ” button. If SCJ can be seen (positive SCJ), VIA test can begin. After applying 3–5% acetic acid solution to the cervix and waiting for 60 s, users will have to click on the camera button again and upload or capture the second cervical image (after VIA test). Then, same as before, the users will have to manually crop the image. The resulting image will be sent to the AI model and within seconds users can see the AI prediction result (Fig. 2).

In the verifier’s dashboard, the app will first display all the unverified patient’s data. The verifier can select the images they prefer, then the app will show certain information regarding that patient (suspicion of cancer, presence of SCJ, and VIA results), along with the cervical images. The verifier will then have to answer 3 questions: (1) Is there any suspicion of cancer? (2) Is the SCJ present? and (3) Is VIA result negative or positive?. For security reasons, the patient’s personal identification information will not be displayed in the verifier dashboard. All data will be safely stored in the hosted cloud server.

### Results

There were 199 women included in this study. All patients were married, aged 30–50 years, and asymptomatic. Out of the 199 data, 134 were used as train-validation data (26 positive VIA and 108 negative VIA results), the remaining 65 data were used as test data.

Based on oncologist evaluation of the test set, 10 patients (15.4%) had positive VIA results and the remaining 55 (84.6%) had negative VIA results. The trained AI model predicted 10 positive VIA results and 55 negative VIA results, consisting of 8 true positives, 2 false positive, 53 true negatives, and 2 false negatives; generating a sensitivity of 80%, specificity of 96.4%, accuracy of 93.8%, precision of 80%, and receiver operating characteristic (ROC) curve with area under the curve (AUC) of. 0.85 (95% CI 0.66–1.0). The classification threshold value used was 0.29.

**Fig. 3:**
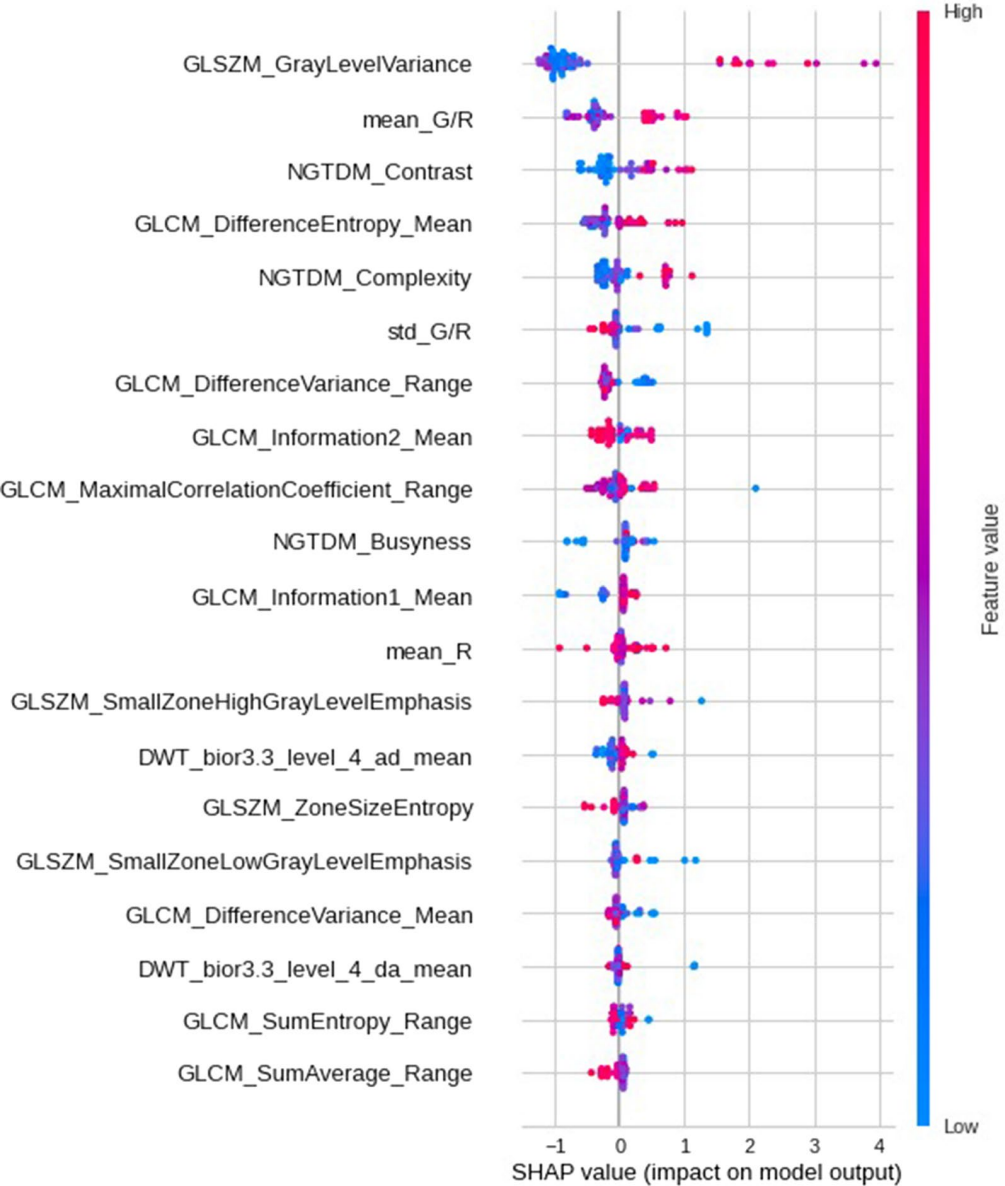
SHAP variable importance plot

To better understand how the model works, we also looked at the SHAP variable importance plot [17] (Fig. 3). This plot can show the positive and negative relationships of the predictors with the target variable. The x axis shows whether the effect of that value is associated with a higher or lower prediction confidence score. The y axis of the plot corresponds to the feature names used by the model, which are sorted descendingly based on the importance towards the model output. Color shows whether that particular variable has a high (in red) or low (in blue) value. As seen in Fig. 3, our model relies the most on GLSZM_GrayLevelVariance feature. A high level of the “GLSZM_GrayLevelVariance” value has a high and positive impact on the VIA prediction score. The “high” comes from the red color, and the “positive” impact is shown on the X-axis. Similarly, we will say the “std_G/R” is negatively correlated with the target variable.

### Discussion

In this project, we developed an AI model that can instantly determine VIA results. Our model generated sufficient sensitivity, specificity, accuracy, precision, and ROC/AUC. The findings of our study suggested that the developed AI model could aid cervical cancer screening in low resource settings by acting as a decision support system.

Image processing-based methods, a subfield in AI, had been widely used to aid medical evaluations [8]. The performance of image processing algorithms in predicting VIA test results has been widely studied [8, 15, 16, 18, 19]. Previous works demonstrated that automated interpretation of VIA results from colposcopic images generated promising results [18, 19]. However, colposcopy is not practical in remote and low resource settings as it requires a bulky device, a constant maintenance, not easily accessible, and not affordable. Cervicography, a photographic method that permits archive and study of cervical image, has been widely used as an adjunct to VIA test. One study by Hu, et al. developed a deep learning-based visual evaluation algorithm that can recognize cervical precancer. However this work relied on images taken by film camera technique (which has been discontinued), rather than contemporary devices like smartphones [20].

Following recent technological advancements, most smartphones are now equipped with high definition camera, therefore offering a cost-effective, rapid, and non-invasive imaging modality. Cervicography using an easily accessible smartphones becomes a very promising choice in low resource settings. One previous study by Bae, et al. implemented a machine learning algorithm for smartphone-based endoscopic VIA screening. The study utilized an endoscopic probe and smartphone for obtaining cervical image of VIA test. However, the algorithm used in that particular study can only be performed on a computer, hence limiting the practicability [8]. Our study tried to develop an algorithm that can be implemented on a smartphone to support more practical uses. Another study by Kudva, et al. proposed an algorithm for analysis of cervix images acquired using an Android device. In this study, we also developed an algorithm that process smartphone acquired cervical images to instantly predict VIA test results. However, we further packaged this algorithm into a smartphone application that can be widely available to healthcare workers around Indonesia. One strength of our study is that it was conducted on a real screening context with population of previously poorly screened population that we hope may resemble actual target screening population in low- and middle-income countries.

### Conclusion

The AI model developed in this work had sufficient sensitivity, specificity, accuracy, precision, and ROC/AUC. This study demonstrated that the developed AI-based application may potentially be useful in aiding VIA test and overall cervical cancer screening, especially in low resource settings.

## Limitations

Much improvement is still needed on our work before it can reach its practical potential. First, the performance metrics of our algorithm were calculated using a small number of samples. Increasing sampling size could provide a greater number of data for training and testing, which could lead to a much more reliable classification results. Second, the ground truth used here was oncologist evaluation. Training the algorithm using strictly defined cases of precancer, optimally histologically proven CIN2 + , will be more valuable for broader use.

## Data Availability

The datasets used and/or analysed during the current study are available from the corresponding author on reasonable request.

## Abbreviations

AI: Artificial intelligence
AUC: Area under the curve
DWT: Discrete wavelet transform
LBP: Local binary pattern
GLCM: Gray Level Co-occurence Matrix
GLSZM: Gray Level Size Zone Matrix
GMM: Gaussian mixture model
NGTDM: Neighbourhood gray tone difference matrix
ROC: Receiver operating characteristic
ROI: Region of Interest
SCJ: Squamocolumnar junction
VIA: Visual inspection of cervix after acetic acid application
